# Evidence-Based Clinical Algorithm for Hypotonia Assessment: To Pardon the Errs

**DOI:** 10.1155/2018/8967572

**Published:** 2018-04-24

**Authors:** Pragashnie Govender, Robin Wendy Elizabeth Joubert

**Affiliations:** School of Health Sciences, University of KwaZulu-Natal, Westville Campus, Private Bag X54001, Durban 4000, South Africa

## Abstract

Despite the many advances in diagnostics, the clinical assessment of children with hypotonia presents a diagnostic challenge for clinicians due to the current subjectivity of the initial clinical assessment. The aim of this paper is to report on an evidence-based clinical algorithm (EBCA) that was developed for the clinical assessment of hypotonia in children as part of the output of a multiphased study towards assisting clinicians in more accurate assessments. This study formed part of a larger advanced mixed methods design. The preceding phases of the study included a systematic review, a survey amongst clinicians, a consensus process (Delphi technique), and a qualitative critique with multiple focus groups. Samples were drawn from three professional groups (occupational therapists, physiotherapists, and paediatricians). Data were analysed at each stage and merged in the development of the EBCA. The EBCA followed a rigorous process of development and critique. The methods for formulating changes in the revision and development of the EBCA are presented together with a description and presentation of the final algorithm for practice. The overarching concepts that guided the development and refinement of the EBCA are described, taking into consideration knowledge translation, evidence-based practice, and the value of EBCAs in addition to recommendations for stakeholder uptake. The EBCA is envisaged to be useful in practice for clinicians who are faced with the assessment of a child that is suspected as having hypotonia via a systematic process in identifying specific characteristics that are associated with low muscle tone.

## 1. Introduction

### 1.1. To Err Is Human

With the advent of this millennium came the realisation that* to err is human*. The Institute of Medicine's (IOM) provocative report in 2000 [1] fundamentally changed the course of how the world configured health and quality of care. Altman et al. [2] characterised this report, at the time, as the most influential health care publication in the preceding two decades. Now, a decade and half has since passed and there have been tremendous efforts at improving health care internationally. Fortunately, this has been no different in the field of child neurology. In the last two decades, child neurology has experienced significant progress, especially within the fields of genetics, molecular neurobiology, and neuroimaging diagnostic techniques [3]. As this IOM report [1] sparked issues around medical errors and patient safety, the paediatric scientific community continued to work towards efforts of early detection and early intervention in children with neurological conditions to ensure the quality of care as advocated for by this report.

### 1.2. Advocating for Accurate Assessment towards Quality Care in Hypotonia

In this paper, the authors report on attempts in ensuring this quality of care and in advocating for a reduction in clinical assessment error in children who present with hypotonia, a symptom of an array of neurological and genetic conditions. Despite the many advances in diagnostics, the clinical assessment of infants with the symptom of hypotonia cannot be overstated. The hypotonic child often presents as a diagnostic challenge for clinicians [4, 5] due to the current subjectivity of the initial clinical assessment of hypotonia. Establishing an accurate diagnosis is thus essential in prognosis and management and in appropriate intervention strategies. Differentiating the likely causes of hypotonia is important in sparing some infants and children from invasive diagnostic tests such as muscle biopsies. In genetic cases it is important to reach the accurate diagnosis so that counseling may be undertaken. The array of diagnoses to consider is increasingly challenging; the severity is variable and is dependent on the underlying causes and thus a careful and thorough approach is necessary [4–6]. The severity and progression of hypotonia vary with each child and their diagnosis. Notwithstanding the underlying cause and progression, authors of papers on evaluation of hypotonia often reiterate the challenge in its assessment as it may be the sign of both nonthreatening and serious conditions [7, 8]. Authors also indicate that often, despite a thorough workup, a diagnosis cannot be established [5] reiterating that this should however not compromise management. Moreover, the frequency of hypotonia and potential improvements with therapy [9] necessitates the need for more accurate and standardized measures [6]. The future of the floppy infant was cited in literature over the last century [10, 11]. Confusion in terminology related to hypotonia was cited in these papers at that stage and continued to be a contentious diagnosis and somewhat erroneous clinical concept over the ensuing years [12]. Contention over the clinical assessment and use of terms continue to be debated into this century [5, 6, 13–15], leaving the clinical assessment of hypotonia open to scrutiny in addition to potential for errors.

### 1.3. Paradigmatic Proposals and EBCAs towards Reducing Human Error

To revisit this notion of* to err is human*; authors in the last decade have made attempts towards the goal of improving quality of healthcare delivery and towards patient safety. Karsh et al. [16] proposed three broad categories of paradigms towards reducing human error in tackling these goals. These include (i) focussing on reducing healthcare professional errors; (ii) focus on reducing patient injuries; and (iii) focus on the use of evidence-based medicine. The authors in this paper position the possibility of evidence-based clinical algorithms (EBCAs) as an opportunity to respond to aspects (i) and (iii) as described in this study. There is evidence in the literature to indicate that the use of care pathways, algorithms, and practice guidelines, in clinical research, will help in standardizing care and provide the necessary requirements for effective diagnostic and counseling interventions [17, 18]. More recent research [19, 20] reiterates the value of evidence-based standards such as algorithms, care pathways, and clinical practice guidelines in guiding patient care.

### 1.4. Acknowledging the Limitation of Uncertainty in Healthcare

Whilst we embrace this position in reducing error, the concept of uncertainty in healthcare cannot be excluded. Hammond [21] advises that clinicians have lived and will continue to live in situations of complex uncertainty in diagnosis, prognosis, and therapy, undeniably in all aspects of the health care process. Hippocrates highlighted that the “art of medicine” lay in understanding the limits of certainty* (ars longa, vita brevis)* which Van Crevel [22] loosely translates as* “you will never see and treat enough cases to avoid every error in your practice” *[22]. Central to this concept of uncertainty is this search for truth. It is thus important to state at this point the authors' position in this study. We leaned towards ontological pluralism, with the realisation that multiple perspectives about complex phenomena can be true, which in this case was the approach to the clinical assessment of hypotonia in children. We acknowledged reality to be what was* useful and practical* and what* works *[23] and in keeping within a pragmatic interpretive framework. We held the notion that there are multiple routes to knowledge and used the tools of research that reflected both deductive (objective) evidence and inductive (subjective) evidence [23].

### 1.5. Contribution of This Study to Taming This Uncertainty

Research and progress in the area of childhood disability have been seriously lagging, particularly in low- and middle-income countries [28]. Due to the improvements in neonatal care and an associated decrease in perinatal mortality, the number of infants who are at risk for developmental problems is gradually increasing [29, 30]. Despite the gains in reducing child mortality with the advent of the Millennium Development Goals (MDGs), specifically goal four [31], which has now come to an end, the ability to predict developmental outcome is still considered difficult. Part of this is attributed to the characteristics of the developing nervous system because the continuous developmental changes of the brain during infancy and childhood can lead to a disappearance of signs of dysfunction present at an early age. The converse is true where children can be free from signs of dysfunction and deficits become evident with increasing age due to the age-related complexity of neural functions [29]. In this study we thus aimed to allow clinicians, as Watkins [32] expresses, “*to take responsibility not only for handling difficult situations but, in particular, for managing the uncertainties which feature in so many clinical encounters*.” We present the output of a multiphased study in the form of an EBCA towards assisting clinicians towards more accurate decisions in approaching the clinical assessment of hypotonia in children.

## 2. Material and Methods

This study followed an advanced mixed methods design [33]. By using a systematic process of collecting evidence from both the literature and clinicians in the field, the development of an EBCA to aid practitioners in the clinical assessment of hypotonia in children was realised. The preceding phases of this larger study are described in other papers [24–27]. The final product, an evidence-based clinical algorithm for the clinical assessment of hypotonia in children, is presented in this paper. Ethical approval was granted by a Biomedical Ethics Committee prior to commencement.

## 3. Results

**Figure 1:**
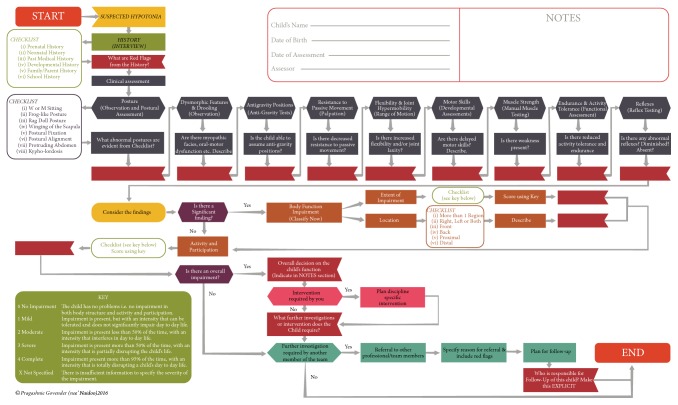
EBCA for the clinical assessment of hypotonia in children.

Figure 1 describes the EBCA following a rigorous process of development and critique. The methods for formulating changes in the revision and development of the EBCA are presented together with a description and presentation of the final algorithm for practice.

### 3.1. Identification of Aspects in the Initial Prototype of the EBCA That Required Revision

Feedback from the critique [27] was used to refine the clinical algorithm presented in preparation for clinical use. Areas that emerged as essential following the critique were centred around inadequacies, misconceptions, and omissions of the algorithm, strengths, clinical use and enablers for implementation, barriers to implementation and resource implications, and appearance and flow as well as general recommendations. The following was noted.

Issues around what constitutes abnormality, the concept of hypotonia with or without weakness, and the extent and location of tone were indicated as misconceptions. Intervention loops, hypotonia terminology, and quantification (descriptors) were raised as misleading aspects. It also emerged that the provision of a guideline may assist clinicians with a wide array of experience. The use of a universal terminology was reiterated to aid communication. Clarity was required as to whether the EBCA forms part of a “clinician's toolkit”; there remained some issues around the unfamiliarity with the ICF taxonomy, and questions were raised around how clinicians will be orientated or trained in the use of the algorithm. With respect to flow and appearance, clinicians questioned what constituted a critical finding, suggesting the use of the words as misleading. Requests for simplified terminology, descriptions of terms, and explanations for the more inexperienced clinicians were also indicated. In terms of the design aspects, inclusion of colour was encouraged (although initially restricted to monochrome due to the resource implications of a polychrome EBCA). Arrows were requested to be bolder to stand out and a simpler flow was encouraged so as to not confuse the assessor. Enablers included having the algorithm remain as a paper-based option as opposed to an electronic option with a pocket and larger version being available for use.

### 3.2. Technical Report and Revisions to the EBCA

In an attempt to address some of the issues raised by clinicians in stage five (critique of the EBCA), a technical report to accompany the EBCA was deemed necessary. A summary of aspects included in the technical report is presented in Table 1. Guidelines are disseminated to encourage high quality care and not as a means towards establishing an identity for a particular professional group or specialty nor are they expected to be exclusionary. Thus, the technical report that accompanies the algorithm clarifies the scope and purpose for intended users, in addition to stakeholder involvement and the development process.

In order to address the issues around the clarity of presentation, the following was ensured [refer to Figure 1]. Descriptors have been included (with a prompt to the key with explanations) within the algorithm ranging from “no impairment” to “complete” with the inclusion of a “not specified” category, as described in the ICF [34]. Intervention loops have been rearranged to address the confusion that had been experienced in the prototype. These have clear monodirectional options. Issues around terminology have been addressed by the inclusion of a glossary within the technical report as well as a change from “critical finding” to “significant finding” that appeared misleading in the initial prototype. Additional process boxes have been included under each of the clinical criteria with a prompt (question) as well as space to document the findings. A section for notes and identifying data of the child is presented. Red flags are prompted throughout the algorithm. Arrows have been amended to flow through each process (also included for checklist boxes) so that the user is not lost (given that the ISO norms for development of algorithms have been adhered to).

Use of colour and coding has been included to visually aid the user. Arrows have been made bolder and checklist boxes have been positioned differently to look more aesthetically pleasing and less confusing. The algorithm has been formatted for legible print on both A4 and larger options.

### 3.3. Progression of the Revised EBCA

From the symbol START, a* history* via an interview is indicated with a checklist flowing through a prompt for identification of red flags from the history taking. The process then flows into a continuous loop for the clinical characteristics beginning with* posture* and ending with* reflexes*. As part of the progression, a question to aid a decision has been included to prompt the assessor, and a red flag box is available for the result to be inserted. Within each of the presented characteristics, the* preferred test and method* are included in parenthesis. In the initial prototype the algorithm proceeded with clinical characteristics that were represented within decision symbols in which a binary decision* (yes or no)* had to be made. Following the critique it was evident that, in order for the process to be complete and comprehensive, a clinician should go through each of the items (in no order of priority) and hence these are represented linearly as opposed to hierarchically (initial prototype). Following the assessment of specific criteria and tests, the process flows to a process symbol, prompting a consideration of findings and rating of* body structure impairment*. At this stage the clinician is able to then quantify the* extent of the impairment* and* location*, indicated as process boxes with the inclusion of the key to aid the decision. There are additional process boxes prompting the user to describe these further. This then leads into the* activity and participation* decision symbol with a checklist and prompt to utilize the key that has been provided. The user is prompted to indicate whether there is an* overall impairment* in a binary decision box. If* yes*, the user makes a decision as to whether discipline specific intervention is required and is returned to the red flag box that considers a team approach by consideration of what* other investigations or interventions* may be required. If a referral is recommended, the user is prompted to provide details including identified red flags and a plan for follow-up. If* no, the process *ends.

## 4. Discussion

Several conceptual aspects have informed our efforts in the development of the EBCA that is described in this paper. Firstly, the authors recognise the knowledge translation (KT) gap that exists between the current care that children receive (assessment of hypotonia) and the evidence available to guide the processes involved in this care [24]. Graham et al. [35] postulated that often patients are denied interventions of proven benefit due to the time taken for research to be incorporated into practice, a sentiment also echoed by researchers in developing countries [36]. In this study, the authors were cognisant of knowledge translation (KT) as being an effective process in closing the gap between researchers and practitioners, knowing full well that innovative approaches to KT designed for different environments are emerging to address evolving knowledge about special clinical populations. As such this study may be a starting point towards KT in an acceptable and user-friendly manner in the clinical field of paediatrics in the form of an EBCA for practice in hypotonia assessment. Whilst practitioners were involved in the development of this algorithm, it may prove useful for inclusion of parents and caregivers in the appraisal of the algorithm for comment on the child's participation in daily occupations as well as on daily routines that are influenced by hypotonia.

Secondly, embracing evidence-based healthcare was essential in the development of this EBCA whilst also realising the value of practitioner knowledge. Although evidence-based healthcare has gained acceptance globally, it remains complex and is sometimes misunderstood. Pearson et al. [37] suggest that sources of evidence accessed by practitioners, regardless of its nature (numerical, qualitative, or anecdotal) or its focus (feasibility, appropriateness, meaningfulness, or effectiveness) inevitably influences healthcare practice in all disciplines. In this study, the authors relied on both the available scientific evidence and practitioner knowledge in contributing to the EBCA for hypotonia assessment. The etymology of the word “evidence” is rooted in the concept of experience, relating to what is apparent and obvious [38]. Thus nonpropositional knowledge or tacit knowledge of professionals [38–40] in addition to propositional knowledge derived from research and scholarship [41] was combined in this study. Inevitably, the authors' attempts have thus resulted in a broader evidence base for practice in the assessment of hypotonia, being realised by an EBCA that has been coherently and sensibly merged to work in the real time of practice. Evidence from a consensus process was embedded within the algorithm with links between recommendations and supporting evidence being explicitly stated in each of the stages of the study to demonstrate the practitioner inputs.

Thirdly, the authors acknowledge that although algorithms are widely used to display logic of diagnosis and management, they have not been taken seriously as a clinical practice standard [18, 19]. Formulation of evidence-based algorithms are said to be an increasing practice in both scientific papers and text books; however their usefulness is questioned, as many of the authors are found not to adhere to the formal requirements [42, 43]. Within this study, the authors have attempted to maintain the basic formal requirements. It has been suggested that methods for writing clinical algorithms that represent expert consensus be sought in practice. This has been fulfilled within this study, as a Delphi process in stage three had assisted in reducing the clinical characteristics and tests initially identified [25] via an expert panel [26]. Thus, items (e.g., clinical characteristics and methods and tests) that appear within the algorithm have not been randomly assigned but are rather the outcome of a previous rigorous phase [26, 27]. Seeking agreement amongst clinicians should be seen as a starting point for establishing criteria that are likely to have significant clinical sensibility and that can be tested to ensure validity. As clinical experience evolves, the opinions of experts may also change, together with their assessment and diagnostic practices. The development of methods for consensus should therefore take cognisance of this and be flexible so that the criteria may be reexamined and revised at intervals [35], possibly with a two-year gap, as new information and research is developed. In ensuring this, stage five involved a critique of the algorithm as an additional attempt to review inclusion of items within the algorithm. These processes within this particular study have all served to ensure robustness in the development of the EBCA presented in this paper.

Finally, this discussion would be incomplete without describing how this effort of the development of an EBCA for hypotonia assessment has been considered with due consideration of stakeholder uptake. Brehaut et al. [44] suggest that an understanding of the cognitive and social processes that affect KT is essential whilst Rogers [45] provides a model for diffusion of the innovation. With due cognisance of these issues and concepts that may arise, the authors have attempted to use feedback from the critique to influence more positive stakeholder uptake. These were with respect to the EBCA (as the innovation) itself, by ensuring clarity of presentation, by the potential communication channels and nomenclature, by the introduction of the technical report to augment the EBCA and by the social system, with consideration of the resource implications. Aside from these initial efforts, a number of additional factors would have to be realised prior to the dissemination and adoption of the EBCA in practice as KT issues are site and possibly country specific given the cultural influences and availability of resources. Some of these include determining the knowledge and understanding required by clinicians of the EBCA in order for implementation to occur and to attempt to determine the optimal mechanisms of social interaction of clinicians to best enable an environment to foster knowledge uptake and change. Moreover a challenge would be for the authors to determine clinician motivation for knowledge uptake and to determine factors that may nurture self-motivation, drive for self-excellence, and willingness to implement and sustain the evidence-based options proposed. Organisational factors may include determining the mode of material presentation and content to ensure that all five adopter groups, according to Rogers [45], may be covered in the implementation process.

## 5. Conclusion

This paper reports on an EBCA as an option for more accurate clinical assessment of hypotonia, a symptom of an array of both neurological and genetic diagnoses, in children. As we progressively sensitise towards early examination, evaluation, and intervention programmes, accuracy of examinations becomes essential in order to contribute to cogent decisions for intervention. Moreover ensuring optimal care, in the face of uncertainty in healthcare, sits with the responsibility of every clinician as part of their ethical and moral obligation. As part of this obligation and the call for more accurate assessment, we have responded with the development of an EBCA for practice. This prepackaged action plan may assist in facilitating the execution of more appropriate and comprehensive assessment towards the ultimate goal of correct and early diagnosis and intervention for hypotonia. The EBCA is envisaged to be useful in practice for clinicians who are faced with the assessment of a child that is suspected as having hypotonia. The benefits of such an algorithm include guiding the clinician in following a systematic process in identifying specific characteristics that are associated with low muscle tone; assisting the clinicians in the choice of the methods that will primarily aid the evaluation of the specific characteristic; providing a qualifier checklist that allows the clinician to quantify the degree of impairment; ensuring holistic assessment by the inclusion of collateral information (history) as well as functional limitations (activity and participation); providing an endpoint following assessment, that is, towards intervention or referral to another professional. This study has further provided an appraisal of the literary evidence to assist clinicians in moving towards more evidence-based practice. Research evidence that is rigorously generated, regardless of design, demands due consideration of its quality prior to its utilization in the clinical field. Hence the next logical step would be the assessment of the quality of the EBCA prior to implementation in practice.

## Figures and Tables

**Table 1:** Description of changes made to the initial prototype of the EBCA.

Description of inclusions in technical report & revised algorithm	
Purpose	
Overall objective	
Health Intent	Clinical assessment of hypotonia in children
Expected benefit	More accurate assessment of Hypotonia, with the inclusion of evidenced-based clinical characteristics and methods
Target population	
Age and gender	0–5 years, male and female
Clinical condition	Suspected hypotonia in any genetic, neurological or other conditions
Severity & stage	Variable: initial clinical assessment for diagnostic purposes or assessment/reassessment for interventions
Health question	
Intervention	Assessment
Outcome	Comprehensive approach to clinical assessment
Health context	Acute and specialised centres, hospital and rehabilitation setting, primary health care clinics or community care centres, special school settings, etc.

Stakeholder involvement	
Intended Users	Health practitioners involved in the initial clinical assessment of hypotonia in children for diagnostic and intervention purposes. The intended users of the algorithm and report include *occupational therapists*;* paediatricians and physiotherapists*
Stakeholders involved in development	Given that the population that is to benefit from this clinical algorithm includes children between 0–5 years, who are unable to contribute to the study, opinions were limited to the practitioners that are responsible for the assessment of this target group. Hence, samples involved in the various stages in the development of the algorithm are the same homogenous population for whom the algorithm has been developed; hence it is modelled on the practice experiences of occupational therapists, physiotherapists, and paediatricians
Strategies and methods used to capture views and preferences	Evidence from literature [24] Cross-sectional survey with *n* = 319 Clinicians [25] Two-round Delphi Consensus Process with *n* = 11 experts [26] Multiple focus groups (*n* = 10) with *n* = 59 clinicians [27]

Development process	
Systematic methods used to search for Evidence	Evidence from literature [24] Cross-sectional survey with *n* = 319 clinicians [25] Two-round Delphi Consensus Process with *n* = 11 experts [26] Multiple focus groups (*n* = 10) with *n* = 59 clinicians [27]
Strengths and limitations of body of evidence	The strengths and limitations of the initial systematic review has been documented [24]. The processes of data collection which has contributed to the final EBCA has been explicitly stated in each of the stages
Link between recommendations and supporting evidence	The initial prototype of the EBCA included a description of how the data collected from the preceding phases were processed and used in its development [27]
